# Green tea consumption and risk of esophageal cancer: a meta-analysis of epidemiologic studies

**DOI:** 10.1186/1471-230X-12-165

**Published:** 2012-11-21

**Authors:** Ping Zheng, Hai-ming Zheng, Xing-ming Deng, Yang-de Zhang

**Affiliations:** 1 National Hepatobiliary & Enteric Surgery Research Center of South University, 87 Xiangya Road, Changsha, China; 2 Department of Digestive Endoscopy, Shanghai 6th People’s Hospital, School of Medicine, Shanghai Jiao Tong University, 600 Yishan Road, Shanghai, China

## Abstract

**Background:**

Green tea has shown the role of chemoprevention for cancer. Recently, several studies suggested that green tea intake may have effect on esophageal cancer risk, whereas the results were inconsistent.

**Methods:**

We performed a meta-analysis of all English and Chinese language studies of green tea consumption and esophageal cancer risk indexed in Medline, Embase, the Science Citation Index, the Chinese Biomedical Database and Wanfang Data from 1980 to June 2012. After reviewing each study, extracting data, and evaluating heterogeneity (Chi-square-based Q test and Ι^2^) and publication bias (Begg and Egger test), a meta-analysis was performed to evaluate the association between high/medium/low green tea consumption and non-drinking esophageal cancer risk. Pooled relative risk (RR) or odds ratio (OR) with 95% confidence intervals (CIs) were calculated using the fixed- or random-effect models.

**Results:**

Ten eligible epidemiologic studies including 33731 participants and 3557 cases for esophageal cancer were included. Eight of which were case–control studies, and two were cohort studies. Overall, there were no association between high/medium/low green tea consumption and non-drinking risk of esophageal cancer (High: highest vs non-drinker: RR/OR = 0.76, 95% CI: 0.49 to 1.02. Medium: drinker vs non-drinker: RR/OR = 0.86, 95% CI: 0.70 to 1.03. Low: lowest vs non-drinker: RR/OR = 0.83, 95% CI: 0.58 to 1.08). When stratified analyses according to study design (case–control and cohort studies), country (China and Japan), participates source (population-based and hospital-based case–control), and gender (female and male), there were significant association between high/medium/low green tea consumption and non-drinking risk of esophageal cancer among female (High: RR/OR = 0.32, 95% CI: 0.10 to 0.54. Medium: RR/OR = 0.43, 95% CI: 0.21 to 0.66. Low: RR/OR = 0.45, 95% CI: 0.10 to 0.79), but not the others.

**Conclusions:**

We did not found significant association between green tea consumption and non-drinking esophageal cancer risk, but an evidence of protective effect was observed among female.

## Background

Esophageal cancer is a major concern in the world, ranking the sixth most common cause of cancer mortality [1]. Lifestyles such as cigarettes smoking, alcohol drinking and dietary habits have been suggested to be associated with the carcinogenesis of esophageal cancer [2,3]. Tea is one of the most widely consumed beverages in the world [4]. Tea is divided into three major types: green tea (non-fermented), oolong tea (half-fermented) and black tea (fermented) according to on the manufacturing process. Green tea and its constituents such as epigallocatechin-3 gallate (EGCG), epigallocatechin (EGC) and epicatechin-3 gallate (ECG) have been shown to inhibit tumorigenesis in many animal models [5,6]. There have been a number of epidemiologic studies evaluated the relation between green tea intake and esophageal cancer risk in human, but with different results. Two large case–control studies [7,8] showed the protective effect of green tea intake on esophageal cancer incidence. However, another case–control study including 883 cases showed that people who have more consumption of green tea more susceptible to esophageal cancer [9]. No quantitative attempt has been to summarize the results of studies exploring a possible association between green tea and esophageal cancer. Therefore, we conducted this meta-analysis to examine the association in epidemiologic studies.

## Methods

### Search strategy

The electronic databases, Medline (1966 to June 2012), Embase (1980 to June 2012), the Science Citation Index (1945 to June 2012), the Chinese Biomedical Database (1981 to June 2012) and Wanfang Data (1980 to June 2012) were searched for epidemiologic studies published in English or Chinese of green tea intake in relation to esophageal cancer risk. We used the search terms “tea”, “food”, “diet”, “beverage”, “drinking” or “tea polyphenol” combined with “esophageal”, “oesophageal”, or “esophagus”. Firstly, the title and abstract of identified relevant studies were used to exclude any obviously irrelevant studies. The full-texts and tables of the remaining articles were retrieved and perused to determine the relevancy of the study design and data, according to the inclusion criteria detailed below. Additional studies were identified by screening the reference lists of each relevant study. Furthermore, reviews concerning the relevant topic were retrieved from the above-mentioned databases in order to potentially broaden the search by identifying additional relevant publications from the studies cited in the reviews.

### Inclusion criteria

The following inclusion criteria were used to select relevant studies for the meta-analysis: (a) human studies, not laboratory or animal studies were included; (b) the daily consumption of the natural green tea product, not of green tea extracts or supplements were recorded; (c) the outcome of interest had to be an incidence of esophageal cancer; (d) relative risk (RR) or odds ratio (OR) estimates with corresponding 95% CIs (or sufficient information to calculate them) were reported. If two or more studies used the same population resource or had overlapping subjects, only the study reporting the largest population was selected for inclusion in the meta-analysis.

### Data extraction

Two reviewers (Ping Zheng and Haiming Zheng) independently performed the data extraction. Disagreements were resolved by reviewers (Deng and Zhang), and a consensus was reached for all data prior to meta-analysis. The following information was collected: the first author’s name, publication year, the country of origin, follow-up duration, gender, the number of participants (cases and cohort size), measurements of green tea consumption, relative risk (RR, which is a ratio of the exposed group and non-exposed group incidence rate and suitable for cohort/prospective study), or odds ratio estimates (OR, which is suitable for case–control studies), and their corresponding 95% confidence intervals (95% CIs). We treated them as two different studies when a study provided separate RR/OR estimates for men and women. If a study provided several RR/ORs, we extracted the RR/ORs reflecting the greatest degree of control for potential interaction factors. When a study provided RR/OR for both esophageal cancer and invasive esophageal cancer, we used the former due to getting more cases.

### Statistical analysis

To evaluate the association between green tea consumption and risk of esophageal cancer, the RR/OR with 95% CIs were calculated using pooled data from the studies. Data pooling was carried out by using the fixed effects model (based on the Mantel-Haenszel method) or the random effects model (based on the Dersimonian and Laird method) [10,11] The random effects model was used if heterogeneity existed between the studies from which the data was extracted; otherwise, the fixed effects model was used. Statistical heterogeneity between studies was assessed with the Chi-square-based Q test and Ι^2^, and heterogeneity was considered significant when the two-tailed *P* value was less than 0.10 [12]. Ι^2^ was used to qualify variation in RR/OR that was attributable to heterogeneity [13]. Publication bias was estimated by using the Begg and Mazumdar adjusted rank correlation test and the Egger regression asymmetry test [14,15]. Finally, the statistical significance of the RR/OR was determined by using the Z test.

Since the original data of tea consumption dose is nonlinear, we divided the level of consumption into high, medium and low groups. We calculated the highest/lowest level of tea consumption as high/low group to compare with the non-drinking when the original literature provided the group of tea-drinking dose. When there is no dose group, we use the drink group as highest/lowest level of tea consumption respectively. We calculated the drinking of tea consumption as medium group to compare with the non-drinking when the original literature provided the group of tea-drinking. And when there is several dose groups, we use the combination as drink group. So we generated three group of tea intake: highest vs non-drinker, drinker vs non-drinker, lowest vs non-drinker.

We performed meta-analysis for all the included studies, and then made subgroup analysis according to study design, country, participates source and gender. This work was conducted on the basis of MOOSE guidelines proposed by the Meta-Analysis of Observational Studies in Epidemiology group [16]. All *P* values are two-tailed. For all tests, *P* values < 0.10 are considered statistically significant, except for heterogeneity. All analysis was performed by the Stata version 11.0 software (Stata Corporation, College Station, Texas).

## Results

### Characteristics of included studies

Ten epidemiologic studies [7-9,17-21] including 33731 participants and 3557 cases of esophageal cancer were identified according to the inclusion criteria of the meta-analysis. The characteristics of the included studies are summarized in Table 1. The publication dates in this study ranged between 1994 and 2011. Eight of them [7-9,18-21] were case–control studies (seven conducted in China and one in Iran), and the other two were cohort studies (conducted in Japan) [17]. Among eight case–control studies, seven studies were population-based case–control (PCC) [7-9,18,20,21]. Besides, the other was hospital-based case–control (HCC) [19]. In addition, there were two studies [7,18] provided gender-specific OR estimates and 95% CIs for the association between green tea consumption and esophageal cancer risk.

**Table 1:** Characteristic of including studies of green tea intake and incidence risk of esophageal cancer

Study	Country; design	Study period	Population	Green tea intake levels OR or RR (95% CI)	Adjustments
Gao (1994)	Shanghai China population-based, hostipal-control study	1990-1993	902 (male:622, female: 280) cases and 1552 (male:854, female: 698) hospital-based controls	Male: drinking vs. never drinking 0.80 (0.58-1.09) 1-199g/month vs. never drinking 0.79 (0.53-1.17) >200g/month vs. never drinking 0.79 (0.56-1.13) Female: drinking vs. never drinking 0.50 (0.30-0.83) 1-199g/month vs. never drinking 0.77 (0.39-1.53) >150g/month vs. never drinking 0.34 (0.17-0.69)	Age, education, birthplace, cigarette smoking (both sexes), and alcohol intake (men only).
Yang (1999)	Yangzhong China population-based, case control study	1998-1998	68 cases and 68 population- based controls	drinking vs. never drinking 0.20 (0.06- 0.67)	Age, education, cigarette smoking, and alcohol intake .
Mu (2003)	Taixing, China population-based, case control study	2000-2000	218 cases and 415 hospital based controls	<125g/month vs. never drinking 1.13 (0.67-1.92) 125-250g/month vs. never drinking 0.78 (0.46-1.34) >250g/month vs. never drinking 0.58 (0.35-0.97)	Age, education, cigarette smoking, and alcohol intake .
Astunobu,cohort 1 (2006)	Japan prospective cohort study	9 years	38 cases among 9008 residents in Miyagi prefecture	1-2 cups/day vs. < never or occasionally 0.69(0.17-2.91) 3–4 cups/day vs. < never or occasionally 1.58(0.52-4.76) 5 cups/day vs. < never or occasionally 1.78(0.66-4.82)	Age, sex, cigarette smoking, alcohol consumption, consumption of black tea, coffee
Astunobu,cohort 2 (2006)	Japan prospective cohort s tudy	7.6 years	40 cases among 17715 residents in Miyagi prefecture	1-2 cups/day vs. < never or occasionally 1.22 (0.47-3.19) 3–4 cups/day vs. < never or occasionally 0.85 (0.30-2.40) 5 cups/day vs. < never or occasionally 1.61(0.71-3.66)	Age, sex, cigarette smoking, alcohol consumption, consumption of black tea, coffee
Wang (2007)	China population based case control study	2004-2006	355(male:223, female: 132) cases and 408 (male:252, female: 156) population controls	Male: drinking vs. never drinking 1.37 (0.95–1.98) Female: drinking vs. never drinking 0.26 (0.07–0.94)	Age (continuous), , sex, cancer family history and BMI, marital status and education years. smoking , alcohol drinking.
Wu (2009)	Dafeng China population based case control study	2003-2007	637(male:426, female: 211) cases and 1938 (male:1368, female: 570) Dafeng population controls	<150g/month vs. never drinking 1.0 (0.7-1.3) 125-250g/month vs. never drinking 1.0 (0.6-1.8) >250g/month vs. never drinking 1.0 (0.6–2.0)	Age , gender, education level, income, cancer family history, BMI smoking , alcohol drinking. tea temperature
Wu (2009)	Ganyu China population based case control study	2003-2007	883 (male:765, female: 118) cases and 1941 (male:1548, female: 393) Gany population controls	<150g/month vs. never drinking 1.1(0.7-1.7) 125-250g/month vs. never drinking 1.0 (0.7-1.6) >250g/month vs. never drinking 1.6 (1.1–2.2)	age , gender, education level, income, cancer family history, BMI smoking , alcohol drinking. tea temperature
Chen (2011)	Guangdong China hospital based case control study	2004-2010	150 cases and 300 hospital based controls	<100g/month vs. never drinking 1.27(0.72-1.89) 100-250g/month vs. never drinking 0.97 (0.59-2.56) >250g/month vs. never drinking0.92 (0.49-2.32)	age, sex, education level, annual income, cancer family history, smoking and drinking status
Islami (2009)	Northern Iran population based case control study	2003-2007	266 cases and 386 population- based controls	Daily, weekly vs Never, <weekly 0.65 (0.32 to 1.31)	ethnicity, daily vegetable intake alcohol consumption, tobacco or opium ever use, duration of residence in rural areas,

OR, odds ratio; RR, relative risk; CI, confidence interval.

### Meta-Analysis of categories of consumption

**Figure 1:**
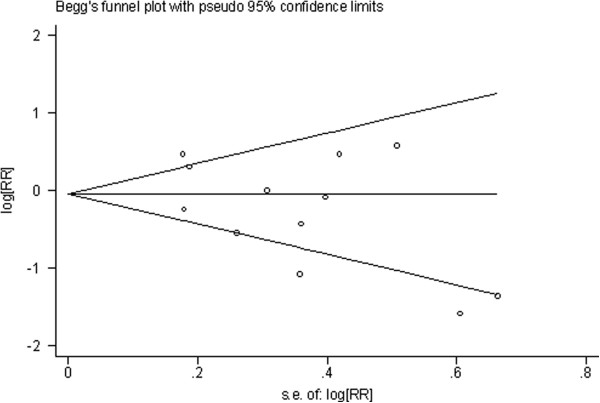
**Begg’s funnel plot of studies on high green tea intake and esophageal cancer risk about results of the included studies.** The solid line in the center is the natural logarithm of pooled relative risk ratio (RR/OR), and two oblique lines are pseudo 95% confidence limits. SE, standard error.

**Figure 2:**
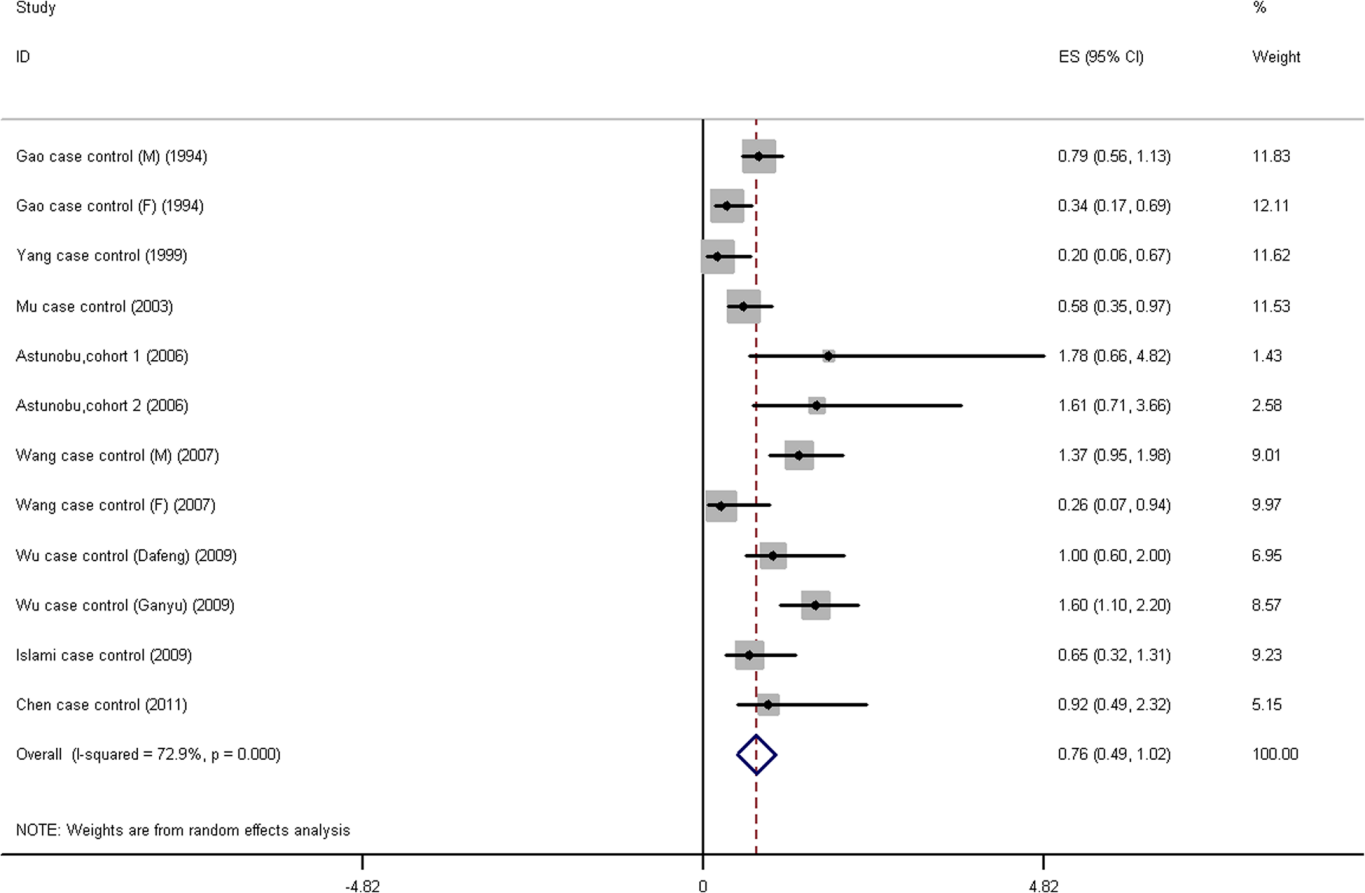
**Forest plot: Results of the studies on high green tea intake.** The size of the data markers (squares) corresponds to the weight of the study in the meta-analysis. The combined relative risk is calculated using the random effects method.

**Figure 3:**
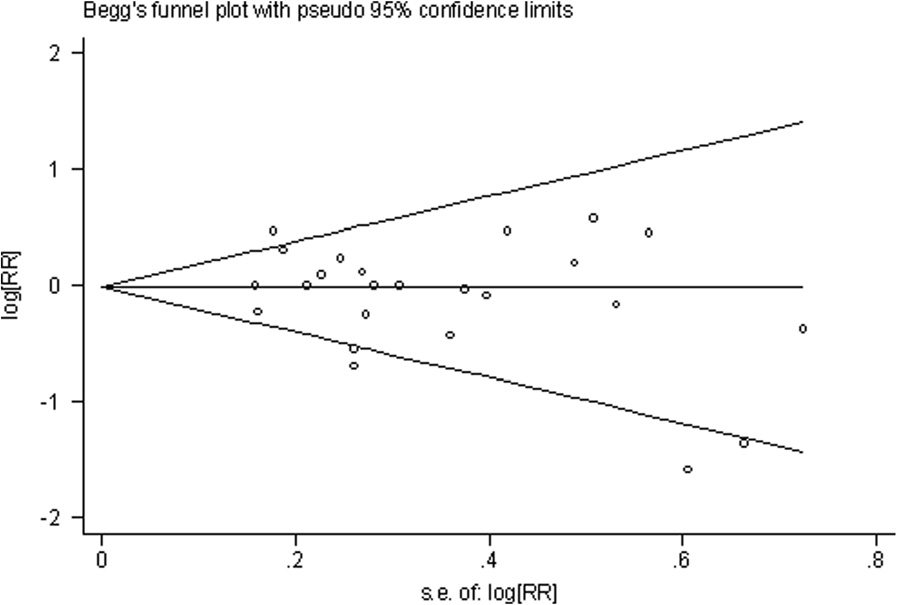
**Begg’s funnel plot of studies on medium green tea intake and esophageal cancer risk about results of the included studies.** The solid line in the center is the natural logarithm of pooled relative risk ratio (RR/OR), and two oblique lines are pseudo 95% confidence limits. SE, standard error.

**Figure 4:**
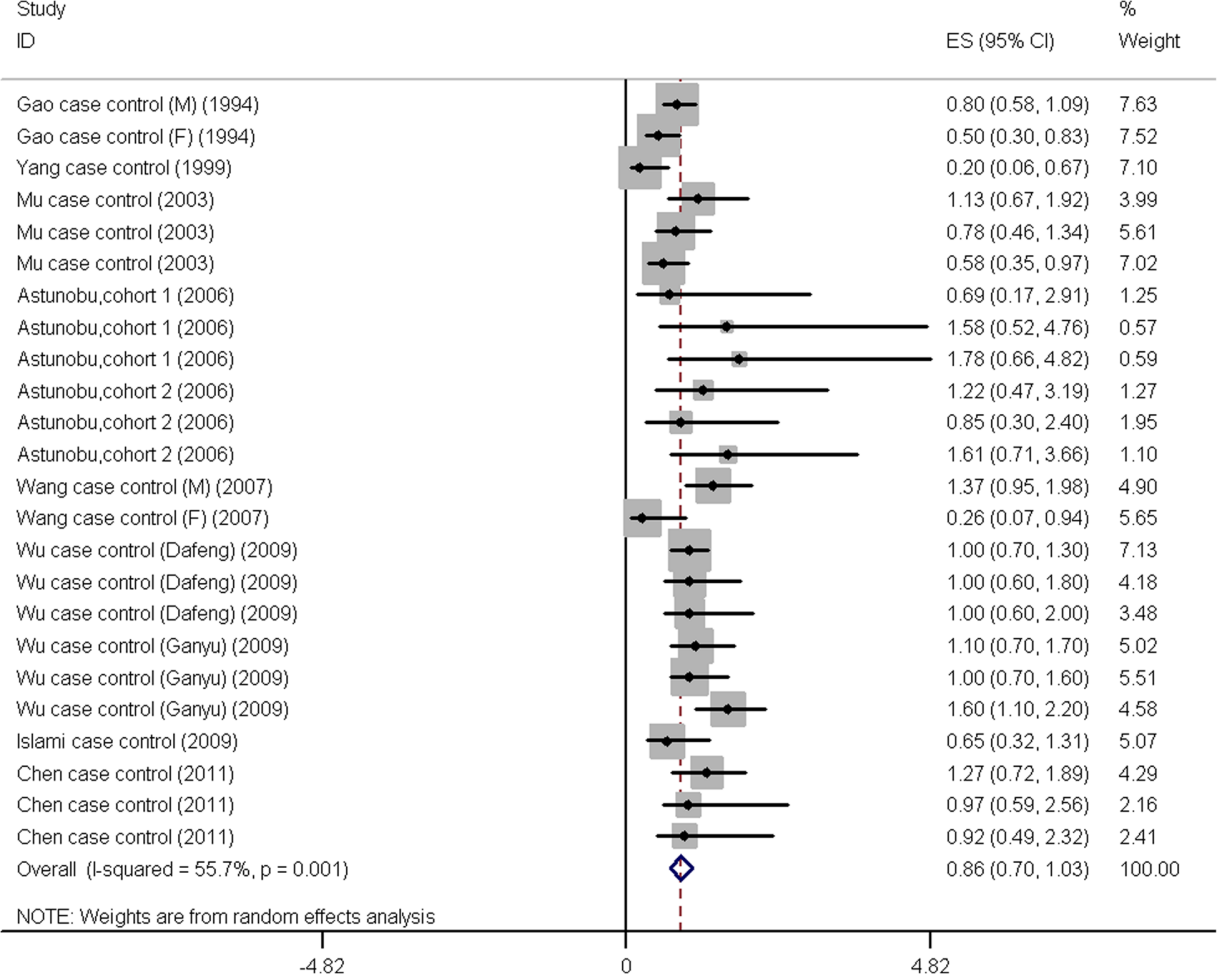
**Forest plot: Results of the studies on medium green tea intake.** The size of the data markers (squares) corresponds to the weight of the study in the meta-analysis. The combined relative risk is calculated using the random effects method.

**Figure 5:**
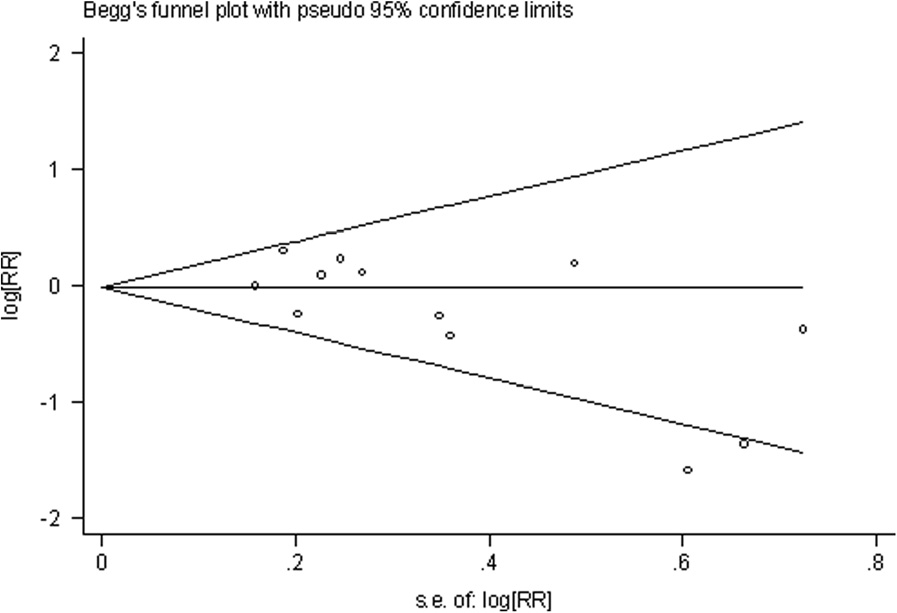
**Begg’s funnel plot of studies on low green tea intake and esophageal cancer risk about results of the included studies.** The solid line in the center is the natural logarithm of pooled relative risk ratio (RR/OR), and two oblique lines are pseudo 95% confidence limits. SE, standard error.

**Figure 6:**
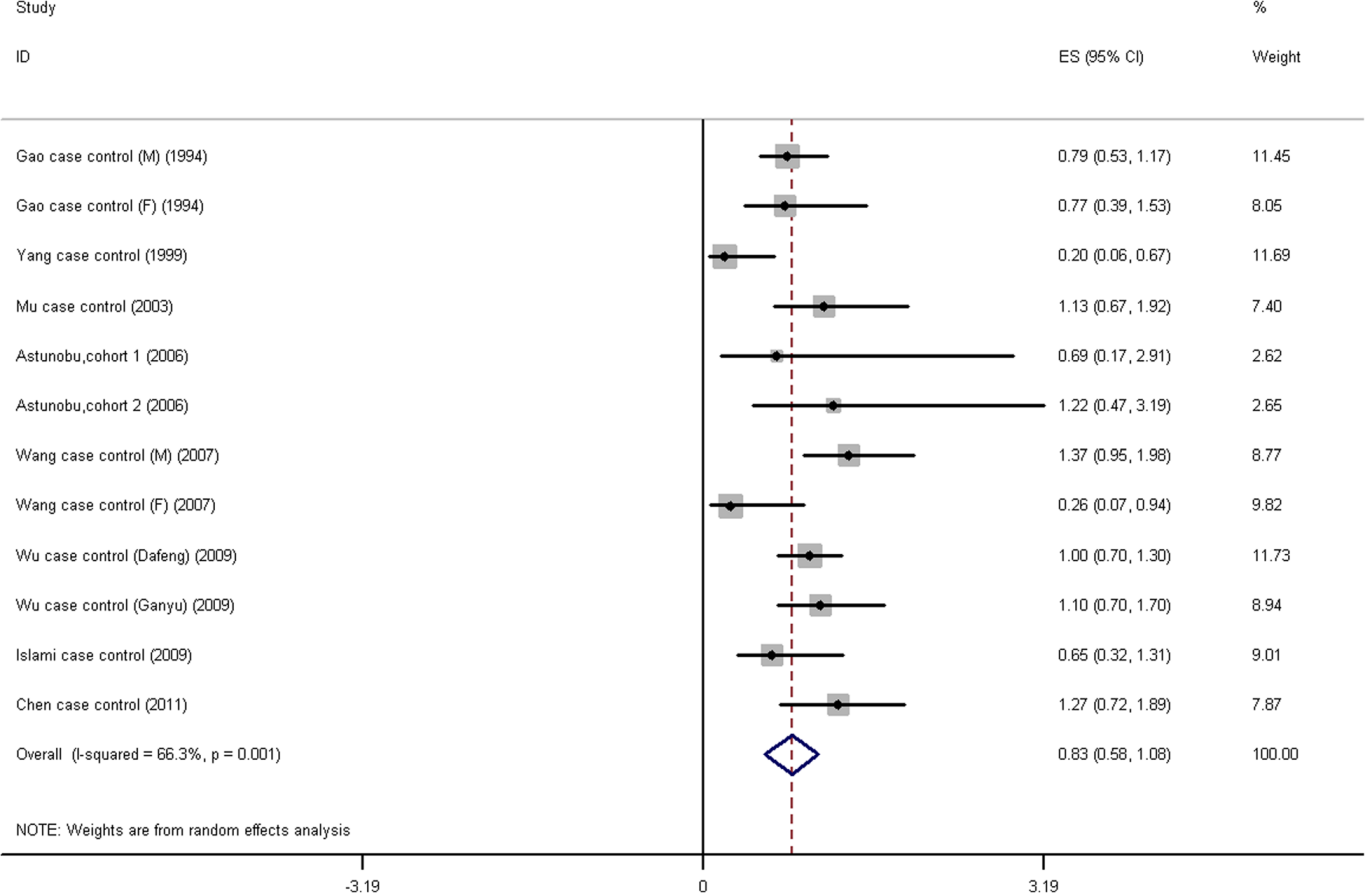
**Forest plot: Results of the studies on low green tea intake.** The size of the data markers (squares) corresponds to the weight of the study in the meta-analysis. The combined relative risk is calculated using the random effects method.

Three categories of consumption have been generated: high (Figure 1, Figure 2, Table 2), medium (Figure 3, Figure 4, Table 3), low (Figure 5, Figure 6, Table 4).

**Table 2:** Risk estimates of high green tea consumption with esophageal cancer by sex, geographic region and type of epidemiologic studies

	No. of studies	No. of cases	RR or OR (95% CI)	Heterogeneity		Publication bias	
				p	I^2^	Begg’s test	Egger’s test
**Overall**	10	3557	0.76(0.49-1.02)	0.00	0.73	0.37	0.16
**Country**							
Japan	2	78	1.67(0.46-2.87)	0.90	0.00	1.00	NA
China	7	3213	0.73(0.44-1.02)	0.00	0.79	0.12	0.05
Northern Iran	1	266	0.65 (0.32-1.31)	NA	NA	NA	NA
**Study design**							
Cohort studies	2	78	1.67(0.46-2.87)	0.90	0.00	1.00	NA
Case control	8	3479	0.72(0.45-0.98)	0.00	0.76	0.07	0.02
**Participates source**							
PCC	7	3329	0.71 (0.43-0.98)	0.00	0.78	0.05	0.02
HCC	1	150	0.92 (0.49-2.32)	NA	NA	NA	NA
**Gender**							
Male	2	845	1.04(0.48-1.60)	0.05	0.73	1.00	NA
Female	2	412	0.32(0.10-0.54)	0.75	0.00	1.00	NA

NA; not applicable; PCC: population based case control; HCC: hospital based case control.

**Table 3:** Risk estimates of medium green tea consumption with esophageal cancer by sex, geographic region and type of epidemiologic studies

	No. of studies	No. of cases	RR or OR (95% CI)	Heterogeneity		Publication bias	
				p	I^2^	Begg’s test	Egger’s test
**Overall**	10	3557	0.86(0.70-1.03)	0.00	0.56	0.24	0.22
**Country**							
Japan	2	78	1.14(0.46-2.87)	0.90	0.00	0.26	0.17
China	7	3213	0.86 (0.67-1.05)	0.00	0.67	0.04	0.03
Northern Iran	1	266	0.65 (0.32-1.31)	NA	NA	NA	NA
**Study design**							
Cohort studies	2	78	1.14(0.46-2.87)	0.90	0.00	0.26	0.17
Case control	8	3479	0.85(0.67-1.02)	0.00	0.65	0.03	0.02
**Participates source**							
PCC	7	3329	0.82(0.63-1.01)	0.00	0.69	0.03	0.01
HCC	1	150	1.13(0.69-1.57)	0.77	0.00	0.30	0.01
**Gender**							
Male	2	845	1.04(0.49-1.59)	0.05	0.74	1.00	NA
Female	2	412	0.43(0.21-0.66)	0.35	0.00	1.00	NA

NA; not applicable; PCC: population based case control; HCC: hospital based case control.

**Table 4:** Risk estimates of low green tea consumption with esophageal cancer by sex, geographic region and type of epidemiologic studies

	No. of studies	No. of cases	RR or OR (95% CI)	Heterogeneity		Publication bias	
				p	I^2^	Begg’s test	Egger’s test
**Overall**	10	3557	0.83(0.58-1.08)	0.00	0.66	0.12	0.03
**Country**							
Japan	2	78	0.96(0.01-1.92)	0.59	0.00	1.00	NA
China	7	3213	0.85(0.56-1.13)	0.00	0.75	0.18	0.03
Northern Iran	1	266	0.65 (0.32-1.31)	NA	NA	NA	NA
**Study design**							
Cohort studies	2	78	0.96 (0.01-1.92)	0.59	0.00	1.00	NA
Case control	8	3479	0.82 (0.56-1.09)	0.00	0.72	0.07	0.01
**Participates source**							
PCC	7	3329	0.78(0.51-1.05)	0.00	0.73	0.03	0.01
HCC	1	150	1.27(0.72-1.89)	NA	NA	NA	NA
**Gender**							
Male	2	845	1.04(0.48-1.61)	0.06	0.72	1.00	NA
Female	2	412	0.45(0.10-0.79)	0.16	0.49	1.00	NA

NA; not applicable; PCC: population based case control; HCC: hospital based case control.

### Meta-Analysis of Case–control studies

Eight case–control studies were included. Of which, two studies [7,18] presented the OR and CIs for female and male respectively, and one study for participates from two different areas. All of them were treated as two studies when analysed.

In the meta-analysis, green tea consumption was found to be associated with a significantly lower risk of esophageal cancer in high group (RR/OR = 0.72, 95% CI: 0.45 to 0.98, Table 2). The *P* value of heterogeneity chi-squared test was < 0.01, and the corresponding I^2^ statistic was 76%, suggesting variability between studies. The *P* values for the Begg’s and the Egger’s tests were *P* = 0.07 and *P* = 0.02, respectively, suggesting the probability of publication bias.

The results in Medium and low group were not statistically significant (respectively: RR/OR = 0.85, 95% CI: 0.67 to 1.02, Table 3. RR/OR = 0.82, 95% CI: 0.56 to 1.09, Table 4). The *P* value of heterogeneity chi-squared test were all < 0.01. The corresponding I^2^ statistic were 65%, 72%. The *P* value for the Begg’s test and Egger’s test were 0.03 and 0.02, 0.07 and 0.01, respectively.

### Meta-Analysis of Cohort studies

Two cohort studies were included [17]. The association between green tea consumption and the risk of esophageal cancer were all not statistically significant (high/medium/low respectively: RR/OR = 1.67, 95% CI: 0.46 to 2.87, Table 2. RR/OR = 1.14, 95% CI: 0.46 to 2.87, Table 3. RR/OR = 0.96, 95% CI: 0.01 to 1.92, Table 4).

The *P* value of heterogeneity chi-squared test were 0.90, 0.90 and 0.59, respectively. The corresponding I^2^ statistic were all 0.0%, indicating a low variability between studies. The *P* value for the Begg’s test and Egger’s test were 1.00 and not applicable, 0.26 and 0.17, 0.07 and 0.01, respectively.

### Combined and Subgroup Analysis

Furthermore, we performed the combined analysis of case–control and cohort studies. The association between green tea consumption and non-drinking risk of esophageal cancer were not statistically significant in three group (High: RR/OR = 0.76, 95% CI: 0.49 to 1.02, Table 2. Medium: RR/OR = 0.86, 95% CI: 0.70 to 1.03, Table 3. Low: RR/OR = 0.83, 95% CI: 0.58 to 1.08, Table 4.). The *P* value of heterogeneity chi-squared test were all < 0.01. The corresponding I^2^ statistic were 73%, 56%, 66%, respectively. The *P* value for the Begg’s test and Egger’s test were 0.37 and 0.16, 0.24 and 0.22, 0.16 and 0.03, respectively. Overall, no association was found between green tea consumption and non-drinking risk of esophageal cancer.

When stratified by country, we did not found association between green tea consumption and non-drinking risk of esophageal cancer in China, Japan and Northern Iran (Tables 2, 3, 4).

When stratified by participates source, we found a significant association between high green tea consumption and non-drinking esophageal cancer risk among PCC (RR/OR = 0.71; 95% CI: 0.43-0.98, *P* < 0.01 for heterogeneity, I^2^ = 78%), but not the HCC (RR/OR = 0.92; 95% CI: 0.49-2.32, with only one study, Table 2). We did not found association between medium/low green tea consumption and non-drinking risk of esophageal cancer in PCC and HCC.

There were two studies [7,18] provided gender-specific RR estimates and 95% CIs for the association between green tea consumption and esophageal cancer risk, therefore we also made stratified analysis by gender. The results of meta-analysis showed that there were significant association between high/medium/low green tea consumption and non-drinking risk of esophageal cancer among female (High: RR/OR = 0.32, 95% CI: 0.10 to 0.54, *P* = 0.75 for heterogeneity, Table 2. Medium: RR/OR = 0.43, 95% CI: 0.21 to 0.66, *P* = 0.35 for heterogeneity, Table 3. Low: RR/OR = 0.45, 95% CI: 0.10 to 0.79, *P* = 0.16 for heterogeneity, Table 4), but not the male.

### Sensitivity analysis

Sensitivity analysis has been carried out by excluding one study from others step by step in each group. They did not alter the original results.

## Discussion

Our meta-analysis of epidemiologic studies did not found significant association between high/medium/low green tea consumption and non-drinking esophageal cancer risk, while an evidence of protective effect was observed among female.

However, there are only two cases of case–control in female studies which existed all in China, this result can lead to selection bias. In addition, the positive results are more easily published, making publication bias generated. All that makes worthy of further consideration about female results. If excluding these factors, some other reason should be considered of the impact among female.

Sex hormone may be an explanation for why female experiencing significantly a lower risk of esophageal cancer when take high level green tea. A sex hormone-mediated pathway may be involved in esophageal carcinogenesis, which was supported by two experimental studies [22,23]. A suppressing effect of estrogen and a promoting effect of androgen were shown in the experimental induction of esophageal cancer by the administration of chemical carcinogen [22]. Meanwhile, the growth rate of metastatic squamous cell carcinoma of the esophagus was inhibited by estrogen and enhanced by testosterone, respectively [23]. Additional studies are warranted to explain and confirm this preliminary evidence.

There have been a number of experimental and clinical studies suggesting drinking beverages at high temperatures to be a cause of esophageal cancer. The facts that more tumors were showed and larger size of esophagus papillomas were rapidly increased when the temperature at 70°C and above was reported by a previous experimental study [24]. In our meta-analysis, two included studies [9,19] both found that drinking tea at high temperature significantly increases risk of esophageal cancer incidence. However, the two studies had different definition for high or normal temperature of green tea drinking, which make it difficult to be stratified for further analysis.

Three studies [9,17,19] included in the meta-analysis have investigated the effects of green tea drinking and dose response relationship. No dose–response relationship was observed among the two studies [17,19]. In the study conducted by Wu [9], higher monthly consumption of tea (*P* for trend = 0.07) and usually drinking tea in high concentration (*P* for trend = 0.01) showed a positive tendency with cancer risk for ever drinker after adjusting for tea temperature. We have analysised data by grouping high/medium/low intake. However, we have not found it is effective between green tea consumption and non-drinking in esophageal cancer risk.

The protective effect of high green tea consumption on esophageal cancer was observed among case–control studies and PCC, but both the heterogeneity and publication bias are significant. So the protective effect among case–control studies and PCC may be incredible. Furthermore we performed the combined analysis of case–control and cohort studies. The heterogeneity and publication bias are not significant in the overall study. So, there may be no significant association between high green tea consumption and non-drinking esophageal cancer risk in the meta-analysis.

Focus on heterogeneity and publication bias of the analyses, we found all the estimates with several studies with a large sample size are very heterogeneous, while estimates with a couple of studies and small sample size have no heterogeneity. For example, both meta-analyses of all studies (ten studies) and case–control studies (eight studies), the *P* for heterogeneity were < 0.01, suggesting variability between studies. However, for meta-analyses among female (two studies) or male (two studies), the *P* for heterogeneity were both > 0.05, suggesting a low variability between studies. When heterogeneity existed we used the random effects model to adjusted. The publication bias among case–control and PCC studies are significant in high group. The cautions for above phenomenon may be as follows: (a) Most of case–control and population-based case–control studies were performed among China [7-9,18-21], and that may cause bias. (b) Retrospective bias. (c) Positive results may be publicated more easily. Furthermore, we performed subgroup analysis by sex, geographic region and type of epidemiologic studies.

However, there are several disadvantages should be considered in our meta-analysis. First, publication bias in China studies or case control studies cannot be missed. The protective effect of green tea in female may be misled by a publication bias because of the female studies all from China studies or case control studies. Second, the epidemiologic studies were not much enough to be stratified for dose and temperature of green tea intake, which may mitigate the result. Third, the non-English and non-Chinese literature could not be reviewed because of the language barrier. Last, most of the studies included in the analysis had been conducted among Asian populations due to popularity of green tea in East Asia. Therefore, the results should be cautious extrapolated to other populations.

## Conclusions

The results of our meta-analysis did not found significant association between green tea consumption and non-drinking esophageal cancer risk, but an evidence of protective effect was observed among female. Additional more studies (especial the cohort studies, and studies from more countries) with careful control of interaction factors including dose and temperature of green tea intake are needed to provide a more definitive conclusion focusing on whether the routine consumption of green tea can guard against esophageal cancer.

## Competing interests

The authors declare that they have no competing interests.

## Authors’ contributions

PZ and HMZ carried out the literature search, selection, validity, assessment, data abstraction and data analysis respectively. PZ and HMZ wrote the paper. XMD and YDZ had the original idea for the paper and revision of the article. All authors reviewed and approved the final draft of the paper.

## Pre-publication history

The pre-publication history for this paper can be accessed here:

http://www.biomedcentral.com/1471-230X/12/165/prepub
